# *Performance Assessment of Emergency Teams and Communication in Trauma Care* (PERFECT checklist)—Explorative analysis, development and validation of the PERFECT checklist: Part of the prospective longitudinal mixed-methods EPPTC trial

**DOI:** 10.1371/journal.pone.0202795

**Published:** 2018-08-24

**Authors:** David Häske, Stefan K. Beckers, Marzellus Hofmann, Rolf Lefering, Christine Preiser, Bernhard Gliwitzky, Paul Alfred Grützner, Ulrich Stöckle, Matthias Münzberg

**Affiliations:** 1 Faculty of Medicine, Eberhard Karls University Tübingen, Tübingen, Germany; 2 DRK Rettungsdienst Reutlingen, Reutlingen, Germany; 3 Department of Anaesthesiology, Faculty of Medicine, University Hospital RWTH, Aachen, Aachen, Germany; 4 Emergency Medical Service, Fire Department, City of Aachen, Aachen, Germany; 5 Faculty of Health, University of Witten/Herdecke, Witten, Germany; 6 Institute for Research in Operative Medicine, University of Witten/Herdecke, Cologne, Germany; 7 Coordination Centre for Health Services Research, University Hospital Tübingen, Tübingen, Germany; 8 Institute of Occupational and Social Medicine and Health Services Research, University Hospital Tübingen, Tübingen, Germany; 9 Megamed Emergency Management GbR, Maikammer, Germany; 10 Department of Trauma and Orthopaedic Surgery, BG Hospital Ludwigshafen, Ludwigshafen, Germany; 11 Department of Traumatology and Reconstructive Surgery, BG Hospital Tübingen, Tübingen, Germany; 12 Centre of interdisciplinary Rescue- and Emergency Medicine, BG Hospital Ludwigshafen, Ludwigshafen, Germany; ESIC Medical College & PGIMSR, INDIA

## Abstract

**Background:**

Trainings in emergency medicine are well structured, but examinations are rarely validated. We are evaluating the impact of pre-hospital emergency trainings on participants and patient care and developed and validated a checklist to assess emergency trainings.

**Methods:**

We used videos recorded at the time points directly before (t0), directly after (t1), and one year after (t2) training to develop the PERFECT checklist (Performance Assessment of Emergency Teams and Communication in Trauma Care). The videos were assessed using semi-qualitative/linguistic analysis as well as expert panel appraisal and recommendations using the Delphi method. The checklist was tested for validity and reliability.

**Results:**

The inter-rater reliability (ICC = 0.99) and internal consistency (α = 0.99) were high. Concurrent validity was moderate to high (r = 0.65 –r = 0.93 (p<0.001)). We included scales for procedures, non-technical skills, technical skills and global performance. The procedures were done faster in the mean over the timeline (t0: 2:29, 95%CI 1:54–3:03 min., t1: 1:11, 95%C 0:53–1:30 min, t2: 1:14, 95%CI 0:56–1:31 min.). All experts rated the recorded scenarios at t0 with the lowest sum score (mean 31±8), with a significantly better performance of the teams at t1 (mean 69±7). The performance at t2 (mean 66 ± 13) was slightly lower than at t1, but still better than at t0. At t1 and t2, linguistic analysis showed a change in the team leaders communication behaviour, which can be interpreted as a surrogate parameter for reduced stress.

**Conclusion:**

The PERFECT checklist has a good validity and high reliability for assessing trauma procedures and teamwork

## Introduction

The care of emergency patients with their countless facets is a great challenge. Non-traumatological patients require a wide range of approaches due to their numerous comorbidities and interactions, whereas due to physiological peculiarities pediatric emergency patients need completely different priorities and strategies in their care. The care for seriously injured patients in turn still deserves considerable attention because a large number of these patients are young, of a working age and the injuries generally have major physical, emotional and socio-economic consequences [1,2]. Today, there is a wide variety of offers for training in acute and emergency care, for example for trauma, resuscitation, pre-hospital or in-hospital emergency situations.

Although these trainings are well structured, in accordance with guidelines, and usually conclude with a written (multiple choice) and practical success evaluation [3–6], the examinations are rarely and not consistently validated [7]. In the study of medicine for the objective assessment of skills and abilities, OSCEs (objective structured clinical examination) are used and these are a valid and reliable tool [8–10].

There are numerous tests for non-technical skills [11] and technical skills [12,13] as well as for trainings or real patient care [14]. Although early experience in OSCE in emergency medicine has been published [15], there is just little literature compared to other medical subjects [16].

### Rationale

In our study, we investigated how an emergency medical service (EMS) system is influenced by systematic training. The reason for a new approach in this EMS was a decreased employee satisfaction and loss of quality in patient care [17]. We knew that the chosen training models have no significant impact on mortality [18], so we chose a prospective longitudinal mixed-methods design, including video analysis of training sequences, to view the impact in its entirety [19]. However, existing assessments or OSCEs were not suitable for verifying changes other than mortality from training, because they do not have the required technical, cognitive and communicative characteristics.

### Specific aims

This article describes the explorative, semi-qualitative development of a checklist for assessment and verification of video analyses of emergency medical trainings. The checklist should enable an objective evaluation and comparability of scenarios or even real trauma patient care by means of point scores.

## Methods

### Context

This analysis was part of the mixed-methods longitudinal EPPTC (Effect of Paramedic Training on Pre-hospital Trauma Care) study evaluating the subjective and objective changes after emergency trainings in participants and real patient care. The complete study is described in the previously published study protocol as well as partial results [19–21].

### Intervention

To improve the pre-hospital care of patients, the medical director of EMS decreed that all of the approximately 300 paramedics had to be trained in PHTLS.

The two-day PHTLS courses teach paramedics and emergency physicians how to improve pre-hospital care for trauma patients [22]. These courses can be regarded as a worldwide standard in the pre-hospital care of seriously injured patients. They use different teaching methods (e.g. lectures, practical case studies, skill training), with a close instructor-participant ratio (1:4), many practical activities and continuous interaction. The courses are conducted by certified instructors (physicians, paramedics, etc.). The priority-based structure ABCDE (Airway, Breathing, Circulation, Disability and Exposure) is taught intensively and practiced in scenario-based training sessions, as well as various skills. The PHTLS statements are similar to the key recommendations of the "German Guideline on Treatment of Patients with Severe and Multiple Injuries" [6].

### Study of the intervention

To assess the impact of the interventions, we chose a mix methods approach. For the longitudinal analysis, we used three measuring points. The first measuring point was just prior to the course (t0), the second measuring point was directly after the course (t1) and the third measuring point was one year after the course (t2). The current publication is concerned with the analysis of videos of the trainings, using qualitative, quantitative and linguistic approaches and with the development of a checklist to assess and to compare the performance of the teams in the training videos in an objective way similar to an OSCE.

### Measurements

We used detailed video analysis for the measurements. For this purpose, three videos were selected at random for each measuring point. To create the scenario-checklist, the videos were analysed, and the results were reviewed, adapted and refined by an expert panel.

The videos were recorded during the trainings for paramedics in context of the overall project (EPPTC-study) [19]. Recording times were at each measuring point. A Panasonic HD Camcorder HC-V100 on a tripod was used for recording and data was stored on SD Memory Cards. The scenarios simulated the pre-hospital care of severely injured patients. Amateur actors represented patients with a leading severe thoracic injury and dislocated ankle fracture, however, always with different causes and stories. Injuries should correspond to an Injury Severity Score (ISS) of approx. 38 (abbreviated injury scale (AIS): AIS 0-0-5-2-3-1). For the present analysis, three videos from each of the three measuring points were randomly selected from the records.

#### Explorative analysis

To gain an impression of the data, we performed an explorative analysis before validation. First, we analysed the timing of the measurements, differentiated according to the three measuring points. Second, we ran a qualitative analysis to develop items for the checklist. Data coding and analyses were performed with the qualitative software program MAXQDA 12 (Berlin, Germany) and followed the methodological concept of a directed qualitative content analysis [23]. Team performance, medical measures, communication characteristics and behaviour were encoded directly into the program. Communication from the team leader to the patient and to the team was transcribed and coded for linguistic analysis.

For linguistic quantitative analysis of the communication between the team leader and team, we used the program "Linguistic Inquiry and Word Count (LIWC)" (Lawrence Erlbaum Associates Inc, Texas). This method categorises word count, sentence punctuation, negation (no, never, not), proportion of words with more than 6 letters (big words), approvals (yes, OK, mmhmm) and first person plural (us, our, us) as well as psychological classifications like positive emotions (happy, handsome, good), anger (hatred, annoying) and cognitive processes (cause, knowledge, effect, perhaps) and fillers [24,25]. The program counts the words in the transcribed text and calculates the percentage of total words that match the specific categories. This analysis did not include other aspects of phonetic language with para-verbal and non-verbal events.

### Expert panel

An expert panel consisting of emergency physicians, medical didactics, sociologists, and human scientists (DH, SB, MH, CP, BG, MM) assessed the results of the video analysis. The Delphi method was used to discuss the codes for the checklist and to discuss the applicability for the practical assessment of scenarios and to reach a final consensus for the determination of the content validity.

Subsequently the inter-rater reliability was tested. Videos of all three time points (t0, t1, t2) were blinded to the time points and then assessed by six experienced instructors (physicians, paramedics with ATLS / PHTLS / ALS instructor level) independently.

### Statistical analysis

To assess the construct validity, we performed a principal component factor analysis (PCA) with varimax rotation. Eigenvalues greater than 0.5 were chosen for the component factors, and factor charges of at least 0.5 were sought for the interpretation of the factor structure.

To calculate the internal consistency of the scales, we used Cronbach’s Alpha. For concurrent validity we correlated the global performance scale, non-technical scale, primary assessment scale and procedure scale.

An inter-rater reliability > 0.8 is recommended [26]. We used the intra-class correlation (ICC), because of continuous variables and more than two raters. Because the evaluation is usually done by a single rater, a two-way random model with single measure ICC (3.1) was used [27].

Pearson coefficient was used to describe interval-scaled correlation. A two-tailed p-value < 0.05 was usually considered as statistically significant. For continuous variables, data is shown as mean ± standard or 95%-confidence interval. For categorical variables, percentages are presented. All data were analysed using the statistical software SPSS (Version 24.0, IBM Inc., Armonk, NY, USA).

### Ethical considerations

The Ethics Committee of the Medical Faculty of the Eberhard Karls University of Tübingen and the University Hospital approved the study proposal, number 197/ 2013BO2. The study is registered in the German Clinical Trials Register with the ID DRKS00004713.

Data collection and analysis were aligned with the data protection officer at the University of Tübingen and the University Hospital of Tübingen. The video recordings were voluntary for the participants and were made after their written consent.

## Results

### Explorative analysis

As part of the qualitative analysis, we ultimately generated 84 codes. The extensive codes gave a differentiated picture of the training but were too complex to use during training or possibly during patient care. Examples of differentiations are the differences between, for example, oxygen applied, oxygen administration ordered, and oxygen administration controlled. During the Delphi process of the Expert Panel, the codes were reduced to items suitable for the checklist.

**Fig 1:**
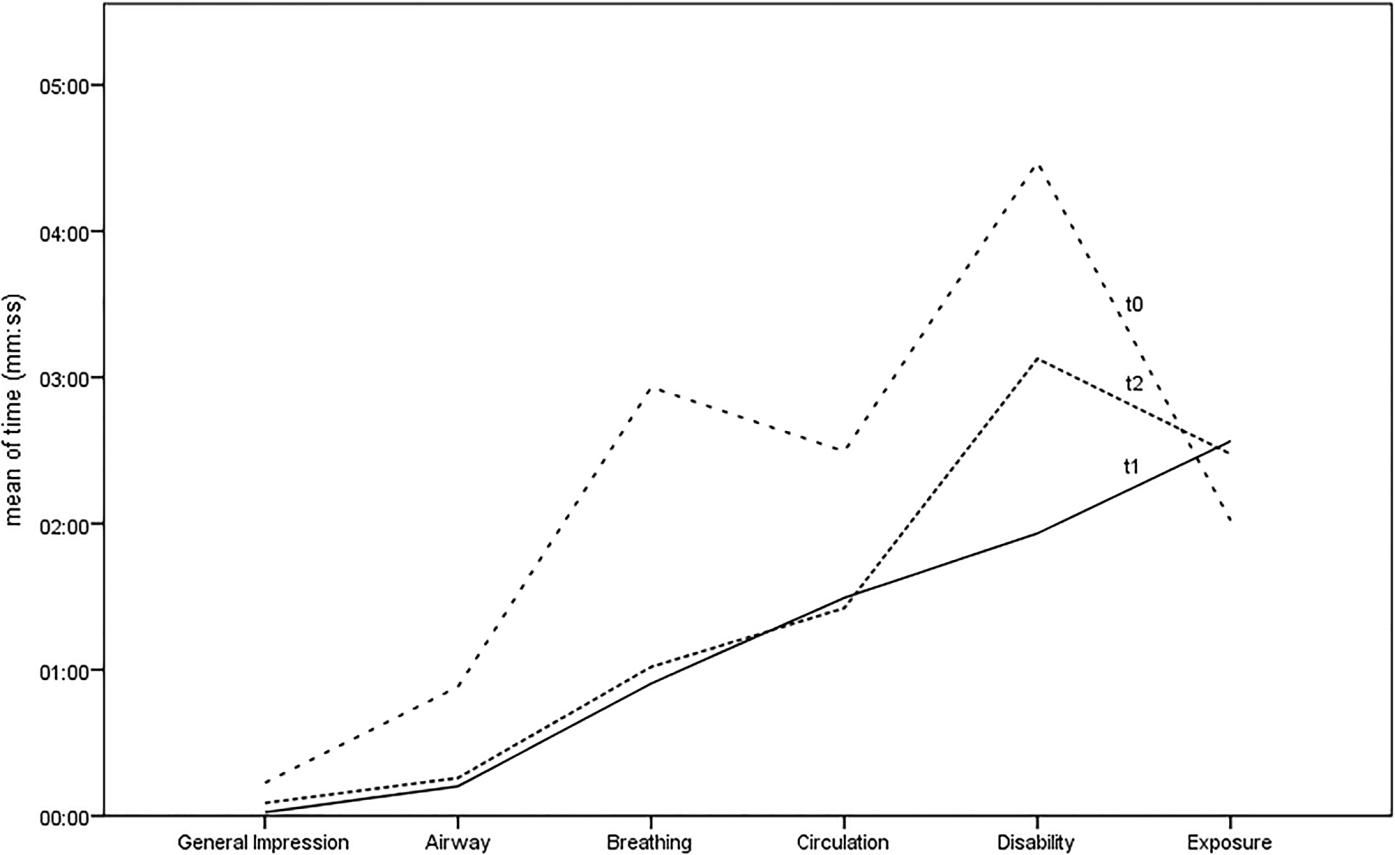
The figure shows the mean time of the measures performed in the primary assessment, grouped according to three different measuring points. The graph t1 fits best the (linear) ABCDE-approach, followed by the graph t2. T0 has the most divergence from the t1.

The chronology of primary assessment showed that most of the procedures were performed earlier at t1 and (t2) as t0 and the confidence intervals became mostly narrower. On average, the procedures were carried out at minute 2:29, 95%CI 1:54–3:03 at t0, at minute 1:11, 95%C 0:53–1:30 at t1 and at minute 1:14, 95%CI 0:56–1:31 at t2. Fig 1 shows the corresponding results with ABCDE approach.

**Fig 2:**
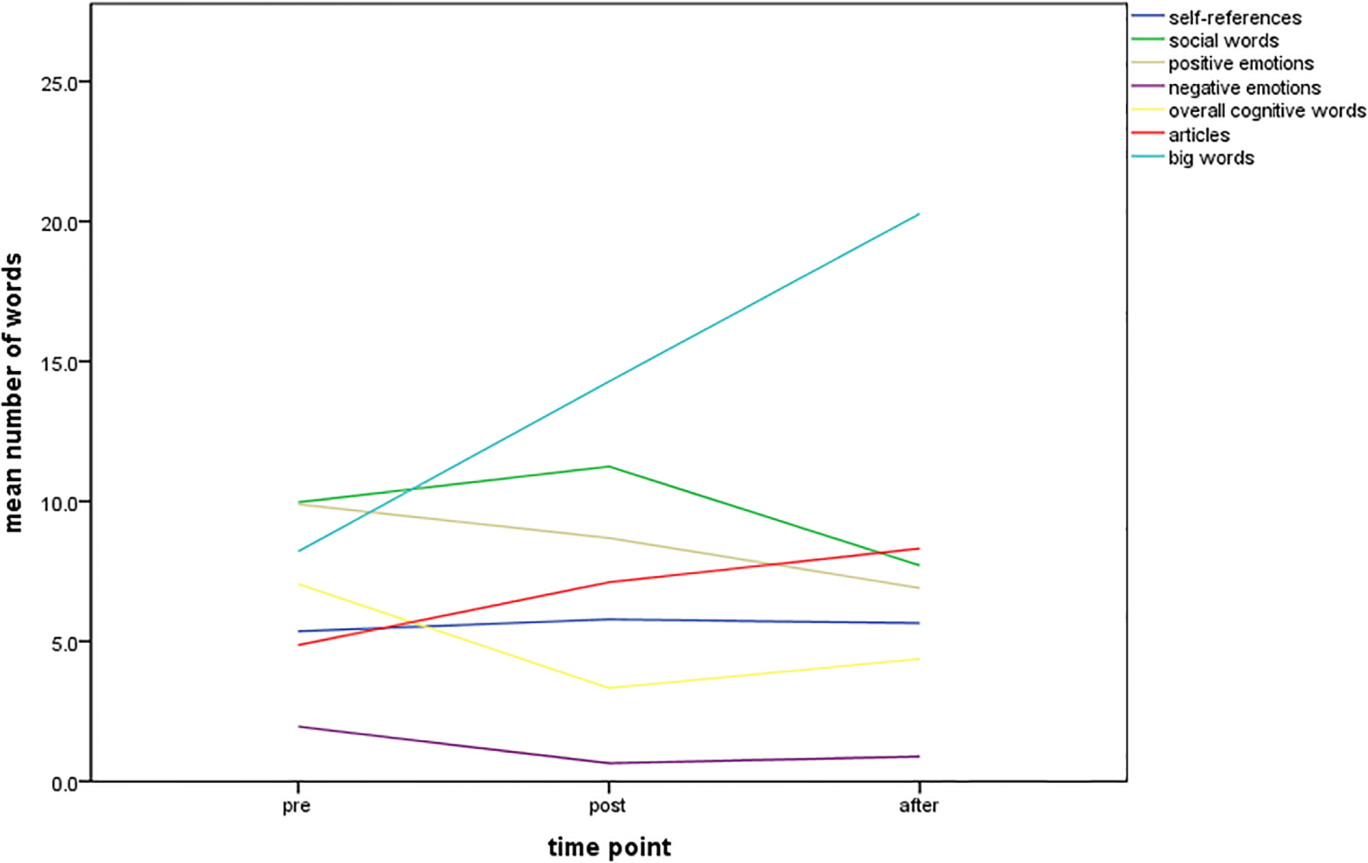
Linguistic analysis of the communication from the team leader to the team, over the three times t0, t1, t2. The increase of big words and articles is obvious, while the cognitive and social words, as well as emotions, decrease.

The linguistic analysis of the communication of the team leader with the team showed a changed communication behaviour from measuring point to measuring point. Fig 2 shows the changes in the different categories. The obvious change is the increase of big words and articles, while the cognitive and social words, as well as emotions, decrease.

### Expert panel

**Fig 3:**
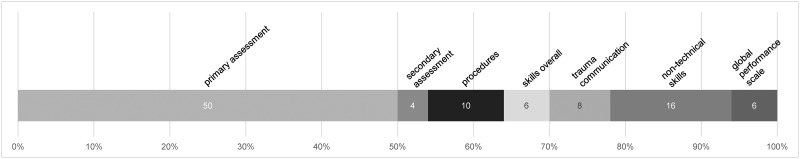
Proportions of the scales in the checklist, based on their maximum points.

Based on the analysis and the expert experience, the checklist *“****Perf****ormance Assessment of* ***E****mergency Teams and* ***C****ommunication in* ***T****rauma Care”* (**PERFECT Checklist**) was created (S1 File). It includes seven scales with a minimum of 6 points and a maximum of 100 points (Fig 3).

The first scale "primary assessment" includes 25 items with the options "application executed" and "timely", which means that each item allows two, overall up to 50 points. The scale "secondary assessment" includes four items with the options "application executed" and "timely". The value of each option was set at 0.5 points, so four points are the possible maximum. Additionally, the time of interventions can be documented.

The expert panel defined a scale "procedures" with five items, in which the most important characteristics for trauma care were defined, which cannot be mapped in other scales. Each item has the value of two points, which means overall twelve points.

The scale "technical skills" includes five skill items and an additional overall item. Each item has a checkbox for "executed" and "indication correct?" as well as a 4-point-performance scale. But only the "skills overall" rating is included in the calculation of the checklist points, with a maximum of six points.

The scale "trauma communication" includes eight items (simply rated) with specific communication points or signal words which were recognised in the qualitative analysis. The maximum is eight points.

The qualitative analysis showed very heterogeneous non-technical skills of the teams. The expert panel chose four items regarding situation awareness and decision-making, leadership and teamwork, workload management and communication. A 4-point performance scale was added, which makes a maximum of 16 points.

Finally, a 6-point "global performance scale" was added to incorporate the experience and judgment of the raters.

### Validity and reliability

**Fig 4:**
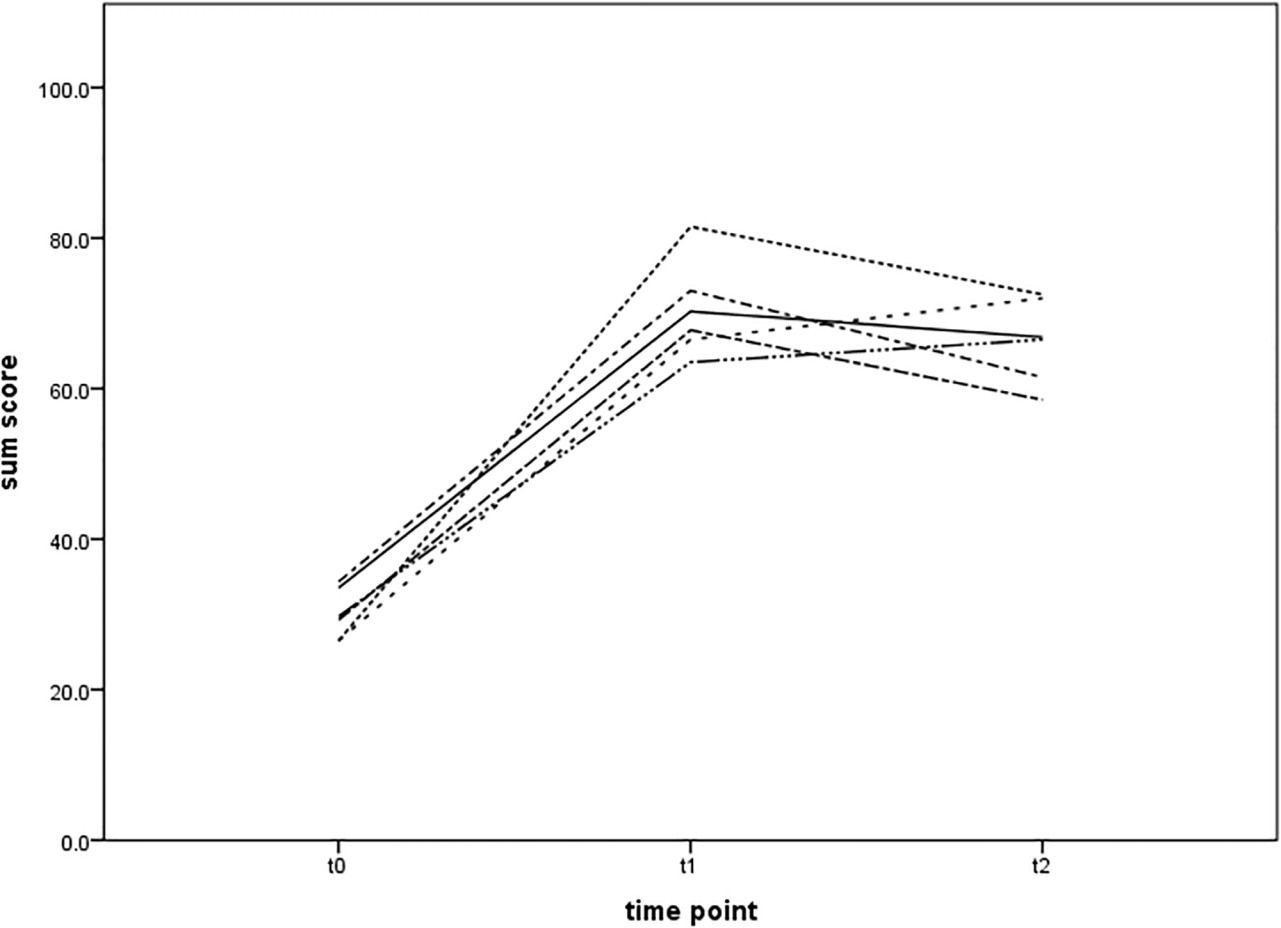
This figure shows the high inter-rater agreement (ICC = 0.993) of the six raters as well as the different appraisal at different measuring points. The evaluation of the reviewers shows a significantly better performance of the teams after the course than before, but with a slight deterioration from right after the course to one year later.

For validation, 36 videos were reviewed by experts as described previously. All experts rated the recorded scenarios at t0 with the lowest sum score (mean 31 ± 8), with a significantly better performance of the teams at t1 (mean 69 ± 7). At t2 the performance was still better (mean 66 ± 13) than at t0, but slightly lower than at t1. This inter-rater agreement is visualised in Fig 4.

### Construct validity

The Kaiser-Meyer-Olkin Measure of sampling adequacy was 0.848, Bartlett's Test of Sphericity was χ^2^ = 207.456 (p<0.001), therefore data showed a good eligibility for PCA.

Three factors with eigenvalues ≥0.5 were extracted by PCA and explained 89.7% of the overall variance. The first factor explained 66.6% of the total variance and included 5 items with factor loadings between 0.83 and 0.92. The second factor explained 14.1% of the variance and comprised just one item with a factor loading of 0.93. The third factor also comprised one item and explained 9.0% of the variance with a factor loading of 0.98. The first factor included the primary assessment, procedures, trauma communication, non-technical skills and the global performance scale and, in accordance with the rating of the experts, explained the most important aspects regarding the quality of simulated trauma care. The second factor included the secondary assessment, which was rarely completed in contrast to the other scales. The third factor was well explained by the technical skills.

### Concurrent validity

The sum score and the sum of the primary assessment showed an excellent correlation (r = 0.916, p<0.001), as well as the sum score with the non-technical skills (r = 0.912, p<0.001) and the sum score with the global rating scale (r = 0.912, p<0.001), shown in Table 1.

**Table 1:** Concurrent validity.

	**Primary assessment**	**Procedures**	**Non-technical skills**	**Global performance scale**
Primary assessment	-			
Procedures	0.654*	-		
Non-technical skills	0.745*	0.825*	-	
Global performance scale	0.806*	0.774*	0.930*	-

* means p<0.001, for two-sided tests. Non-technical skills and global performance scale show the highest correlation (r = 0.930), followed by non-technical skills and procedures (r = 0.825).

### Internal consistency of the scales

Scales with several items have a good consistency: Cronbach’s alpha was 0.93 for the 27-item-scale "primary assessment", 0.87 for the 4-item-scale "secondary assessment", 0.95 for the 6-item-scale "procedures", 0.93 for the 8-item-scale "communication", 0.96 for the 4-item-scale "non-technical skills" and overall 0.99.

### Inter-rater reliability

Inter-rater reliability showed very good values overall and for specific scales. ICC was for the "primary assessment" 0.93, for "secondary assessment" 0.85, for "procedures" 0.93, for the 8-item-scale "communication" 0.93, for "non-technical skills" 0.96 and overall 0.99.

## Discussion

The aim of emergency trainings is to gain assurance manual skills and to act more effective in life-threatening situations by improving the knowledge as well as the structured care of emergency patients. Therefore, cognitive knowledge, technical skills, and procedures with clinical judgment are necessary and elementary for subjective safety in trauma care [28].

A variety of courses are available, but the existing assessments did not match our needs. The PERFECT checklist was developed using qualitative and quantitative analysis and the expertise of experienced academics, clinicians and emergency medicine trainers. The result combines the assessment of (technical) skills, non-technical skills and procedural performance, which are essential for clinical competence in trauma care.

The qualitative analysis gathered data in detail. In the end we separated items in, for example, "indicated", "executed", "executed correctly", "executed without indication". In a second step, we had to decide between analytical details whose clinical relevance may be marginal and practicality of the checklist during scenarios. We chose to focus on clinical aspects and practicality and therefore combined several items in groups with the same clinical relevance, for example we established the item “looked for possible respiratory failure” instead of the detailed items cyanosis, thoracic excursions, breathing work, and diminished tidal volume.

Linguistic analysis was developed in the 1990s to analyse cockpit communication regarding language errors and workload [29]. It resulted in recommendations to keep communication simple by short and clear words, because under increasing stress the brain’s memory capacity decreases to a few seconds [30]. The transferability of the findings to acute medicine is widespread and acknowledged [31,32]. Our linguistic analysis of the communication from the team leader to the team at measuring point t0 to t2 showed an increasing number of words in the categories “big words” and "articles" (Fig 2). The relationships may be multifactorial, but with the knowledge that communication, and respectively speech, becomes shorter and monosyllable under stress [30] these categories can be indicated as surrogate parameters for stress. Stress has a relevant influence on the technical performance, but can be compensated by non-technical skills [33]. In our analysis, we interpreted the increasing number of words in the categories "big words" and "articles" as a surrogate marker, which indicates declined stress levels over the measurement times. This result is also reflected in the assessment of the course participants, as their subjective safety in the care of severely injured patients improved over the measuring points and after the course [28]. Additionally, our "non-technical skills scale" and "primary assessment scale" showed a good correlation with r = 0.745.

In the qualitative analysis of the scenarios, the difference in the non-technical skills of the teams was remarkable. Although the PHTLS system does not provide any NTS teaching content, they have been added to the checklist. For the NTS scale we used a 4-point performance scale, which was labelled and matched with the relevant literature [31,32]. Wallin et al. have also evaluated the impact of training on medical students, with improved clinical skills, but there was no improvement in teamwork [34]. In contrast, in our exploration we saw a significant improvement in teams with mixed experience, but these findings must be confirmed with an adequate sample size.

The checklist developed shows a very good reliability and validity. Our ICC (0.99) is notably higher than the ICC of similar assessment tools (0.6–0.8) [35–37]. The appraisal of the expert panel was in our opinion more important than a calculation of a content validity index, especially because the content validity is always subject to a certain subjectivity and it is strictly speaking not a test quality criterion [26]. Concurrent validity shows high correlation. Along with this, we have established the equally well correlated global performance scale, with which Dankbaar et al. had already had positive experiences [38]. Therefore, it is important to have well-trained users of the checklist.

The different coding of the scales was also discussed in the expert panel. For classical OSCE, binary codes are often used but are at least equal to multi-level rating [39]. Frequently, technical skills tend to use binary rating scales rather than communicative ones [40], and their number of points or range can vary widely [35,37,41]. We decided to use binary coding in the assessments and procedures and used 4-point rating scales for NTS and the skill performance. The global rating scale has a 6-point rating scale.

Qualitative video analysis also showed that some teams met all the points on the scale, but they did not perform in a timely manner, in any structure or at speed, or in terms of rapid identification and management of life-threatening conditions. We also knew that a structured patient treatment has a significant influence on subjective safety in trauma care [28], which is why the expert panel had implemented the scale "procedures" and even weighed their points twice. Thus, the factor can be evaluated once with the two items in the procedure scale, on the other hand, the individual measures have the option of "timely" to note. In addition, the time can be recorded independently of the checklist by the real time measurement.

As described, the PERFECT-checklist was developed to evaluate trainings from PHTLS-courses. In principle, however, it is possible to use this validated checklist for each prehospital trauma training according to a comparable standard. The standard in trauma care in Germany is the "Level 3 guideline on the treatment of patients with severe/multiple injuries" [42]. For PHTLS it is proven that the key recommendations fit the Level 3 guideline [6]. It is to be assumed, but not proven, that other established training programs, such as ITLS, trauma management come to similar statements. Thus, the PERFECT-checklist fits all comparable standards in the acute care of trauma patients.

It should also be possible to use the checklist for real patient care in prehospital setting or in the trauma room as well as for simulation. However, this has not been investigated yet. Even the individual scales of the checklist can be used, whose validation index can be taken from the result part.

## Limitations

The biggest weakness of the checklist is the impossibility to make a differentiated assessment of the skills. This concerns the individual skills (execution, indication, performance, etc.) as well as the embedding into the points system of the checklist to ensure the comparability of scenarios. For example, a scenario with three skills could theoretically receive a triple skill score, while a scenario with only one skill could be very well performed but would have automatically scored less on the one skill. So we decided to include only one overall skill item in the calculation, but to include differentiated skills as a memo for instructors.

Our checklist was developed on scenarios of emergency trainings and not during real patient care and so no statement can be made about how it works in real patient care.

## Conclusion

The importance of a systematic approach instead of a personal approach to team training in high-risk emergency care is crucial [43] and vitally important for improving public health and potentially reducing the mortality of patients. This requires appropriate training, as well as validated opportunities to review long-term training success. With the support of the PERFECT checklist, a validated tool is now available to evaluate trauma trainings with comparable standards.

## Supporting information

S1 File The PERFECT-checklist. (PDF)
