# Supplementary material for: Performance Assessment of Emergency Teams and Communication in Trauma Care (PERFECT checklist)—Explorative analysis, development and validation of the PERFECT checklist: Part of the prospective longitudinal mixed-methods EPPTC trial
Source: PLoS One. 2018 Aug 24;13(8):e0202795. doi: 10.1371/journal.pone.0202795 (PMC6108494; doi:10.1371/journal.pone.0202795)
Supplement: S1 File — (PDF) [file pone.0202795.s001.pdf]

Performance Assessment of Emergency Teams and Communication in Trauma Care
PERFECT-Checklist

Case no:	Assessor:

1.0 Primary assessment			Time	Application performed	Timely
	General impression				
1.		first look/5-second round<br>global overview of the status of the patient		☐1	☐1
2.		recognised kinematics		☐1	☐1
	Airway				
3.		manual cervical spine immobilisation		☐1	☐1
4.		mouth inspection, airway checked		☐1	☐1
5.		necessary measures to optimise and control the airway performed (e.g. airway-manoeuvre, assistant who observed the airway)		☐1	☐1
	Breathing				
6.		respiratory rate stated		☐1	☐1
7.		possible respiratory failure (e.g. cyanosis, thoracic excursions, breathing work, diminished tidal volume) looked for		☐1	☐1
8.		auscultation/breathing sounds stated		☐1	☐1
9.		SpO_2 used		☐1	☐1
10.		necessary measures to optimise oxygenation and support ventilation performed (e.g. oxygen insufflation, ventilation)		☐1	☐1
	Circulation				
11.		pulse checked		☐1	☐1
12.		possible shock signs and hypoperfusion (e.g. skin colour, temperature and moisture, capillary refilling time) looked for		☐1	☐1
13.		bleeding control thorax		☐1	☐1
14.		bleeding control abdomen		☐1	☐1
15.		bleeding control pelvis		☐1	☐1
16.		bleeding control thighs		☐1	☐1
17.		IV lines		☐1	☐1
18.		necessary measures regarding bleeding control performed (e.g. pressure bandage, tourniquet, haemostyptics)		☐1	☐1
19.		necessary measures regarding shock therapy performed (e.g. infusion therapy, tranexamic acid)		☐1	☐1
	Disability				
20.		vigilance checked		☐1	☐1
21.		motor/sensoric responses checked		☐1	☐1
22.		pupils examined		☐1	☐1
23.		treatment appropriate to the D findings		☐1	☐1
	Exposure				
24.		body temperature maintained - recognised temperature		☐1	☐1
25.		further injuries looked for		☐1	☐1

2.0 Secondary assessment		Application	Timely
1.	medical history (SAMPLE) checked	☐0.5	☐0.5
2.	vital signs measured (blood pressure. ECG. e.g.)	☐0.5	☐0.5
3.	continued detailed physical exam performed	☐0.5	☐0.5
4.	further necessary diagnostics and measures performed	☐0.5	☐0.5

Performance Assessment of Emergency Teams and Communication in Trauma Care
PERFECT-Checklist

3.0 Procedures		Application
1.	Team performed a *structured* (ABCDE) approach	☐2
2.	Team did no further harm through unnecessary measures/treatment	☐2
3.	The speed of patient care was appropriate to the patient's condition	☐2
4.	Team performed rapid identification and management of life-threatening conditions	☐2
5.	An appropriate analgesia was performed	☐2

4.0 Skills			Performed	Indication correct?	Performance (duration. positioning. technique) 4=excellent. 3=good. 2=fair. 1=poor
	**Cervical collar**	*Cervical collar was applied in adequate time and manner (cervical collar applied with ≥2 persons)*	☐ yes	☐ yes	☐ 4 ☐ 3 ☐ 2 ☐ 1
	**Immobilisation (e.g. spine board)**	*Immobilisation was performed in adequate time and manner*	☐ yes	☐ yes	☐ 4 ☐ 3 ☐ 2 ☐ 1
	**Tourniquet**	*Tourniquet was applied in adequate time and manner*	☐ yes	☐ yes	☐ 4 ☐ 3 ☐ 2 ☐ 1
	**Thoracic (needle) decompression**	*Thoracic (needle) decompression was performed in adequate time and manner*	☐ yes	☐ yes	☐ 4 ☐ 3 ☐ 2 ☐ 1
	**Pelvic binder**	*Pelvic binder was applied in adequate time and technique*	☐ yes	☐ yes	☐ 4 ☐ 3 ☐ 2 ☐ 1
1.	**Skills overall**	*Overall skills performance*	☐1 necessary skills were mostly performed	☐1 Indication mostly correct	☐ 4 ☐ 3 ☐ 2 ☐ 1

5.0 Trauma communication		Application
1.	potentially critical/non-critical patient stated	☐1
2.	(no) A problem stated	☐1
3.	(no) B problem stated	☐1
4.	(no) C problem stated	☐1
5.	(no) D problem stated	☐1
6.	critical/non-critical patient stated	☐1
7.	10-for10/team-timeout/team information performed	☐1
8.	appropriate trauma centre informed	☐1

6.0 Non-technical skills			Performance 4=excellent. 3=good. 2=fair. 1=poor
1.	**Situation awareness and decision making**	*Team gathers information, anticipates and re-evaluates. Team keeps procedures in mind, adapts to various situations, and allocates attention wisely.*	☐ **4** ☐ **3** ☐ **2** ☐ **1**
2.	**Leadership and teamwork**	*The team leader recognisably leads the team and coordinates the teamwork. Team members follow the leader and complete the task together.*	☐ **4** ☐ **3** ☐ **2** ☐ **1**
3.	**Workload management**	*Team sets priorities dynamically, coordinates activities and follows standards.*	☐ **4** ☐ **3** ☐ **2** ☐ **1**
4.	**Communication**	*Team communicates effectively, anticipates and exchanges information. There are clearly stated plans and intentions.*	☐ **4** ☐ **3** ☐ **2** ☐ **1**

7.0 Global rating scale			
1.			☐ 1 ☐ 2 ☐ 3 ☐ 4 ☐ 5 ☐ 6<br>1=poor 6=excellent
